# Investigating statistical power of differential abundance studies

**DOI:** 10.1371/journal.pone.0318820

**Published:** 2025-04-08

**Authors:** Michael Agronah, Benjamin Bolker

**Affiliations:** 1 Department of Mathematics and Statistics, McMaster University, Hamilton, Ontario, Canada; 2 Department of Biology, McMaster University, Hamilton, Ontario, Canada; University of Idaho, UNITED STATES OF AMERICA

## Abstract

Identifying microbial taxa that differ in abundance between groups (control/treatment, healthy/diseased, etc.) is important for both basic and applied science. As in all scientific research, microbiome studies must have good statistical power to detect taxa with substantially different abundance between treatments; low power leads to poor precision and biased effect size estimates. Several studies have raised concerns about low power in microbiome studies. In this study, we investigate statistical power in differential abundance analysis. In particular, we present a novel approach for estimating the statistical power to detect effects at the level of individual taxa as a function of effect size (fold change) and mean abundance. We analyzed seven real case-control microbiome datasets and developed a novel method for simulating microbiome data. We illustrate how power varies with effect size and mean abundance; our results suggest that typical differential abundance studies are underpowered for detecting changes in individual taxon.

## Introduction

Identifying taxa that show differential abundance between groups holds great potential for clinical applications. For example, a study aimed at assessing the effects of a dietary intervention on microbial composition might analyze the abundance of different microbial taxa between a control group on a standard diet and a treatment group on a gut-health-promoting regimen.

Power analysis allows researchers to determine whether they have a sufficient sample size to detect meaningful effects in their studies. The power of a statistical test is the probability of successfully rejecting the null hypothesis given a particular effect size [1]. Power is determined by the sample size, effect size and the significance threshold (or “alpha level”), as well as methodological factors such as experimental design, number of groups, statistical procedure and model, type of response variable and fraction of missing data.

Power analysis enables researchers to detect meaningful effects and allocate resources efficiently; it aids the reliability and reproducibility of research findings. The primary goal of power analysis is to ensure that a research study has the sensitivity required to detect meaningful effects [1]. Underpowered studies are likely to miss biologically meaningful effects and are more prone to type II errors, which can lead researchers to neglect differences that could be biologically interesting [2]. Even if a low-powered study finds statistically significant results, the estimated effect size will be imprecise [2]. Low power together with a statistical significance filter (for example, only reporting effects with a *p*-value 40*%* ) can lead to overestimation of the true effect (“magnitude”, or type M, error) or an incorrect estimate of the direction of an effect (“sign”, or type S, error) [3].

Microbiome researchers typically focus on three main types of analysis: (1) *analysis of univariate summaries*: reducing the data from each microbiome sample to a single value, such as alpha diversity, and comparing the distribution of these values between groups, (2) *community-wide analyses* using tests such as Permutational Multivariate Analysis of Variance (PERMANOVA) or the Dirichlet-multinomial model to distinguish overall differences in communities, and (3) taxon-by-taxon or *differential abundance* analyses: identifying taxa with biological meaningfully differences between groups. Existing studies on power analysis have focused either on studies comparing univariate (alpha diversity) measures or studies comparing changes in overall microbiome composition between groups [4]. For example, La Rosa et al. [5] developed a reparameterized Dirichlet Multinomial model and a method for estimating the power to detect changes in overall microbial compositions between groups. Kelly et al. [6] proposed a framework for estimating power in PERMANOVA.

To our knowledge, no methods exist for power analysis for differential abundance studies. In practice, every taxon in a microbial community has a different mean abundance and a different effect size (as is typical, we use fold change between groups as effect size in this paper), leading to a different statistical power to detect differences in every taxon. Except for relatively simple analyses, conducting power analysis requires data simulation. Simulating an entire microbial community is challenging because it requires estimating appropriate community-wide distributions for mean abundances and effect sizes of taxa.

Power estimates in a differential abundance study depend on the abundance of individual taxa. For example, effect sizes of taxa with high abundance in both control and treatment groups are more likely to be detected compared to effect sizes of taxa that are rare in both groups. Unlike univariate power analysis (that is, power analysis involving univariate quantities) where one can specify a single value for effect size and power, in a taxon-by-taxon power analysis there are multiple values of effect size and power; one for each taxon. This typically means hundreds or thousands of effect sizes and power values.

Several studies have raised concerns about low power in microbiome studies [7,8]. For example, Kers and Saccenti [7] showed that microbiome studies comparing alpha and beta diversities (PERMANOVA) between groups might be underpowered. The goal of this study is to investigate the issue of potential low power to detect effect sizes of individual taxa within a differential microbiome study. We develop a novel method for simulating microbial communities and estimating power at the level of an individual taxon. Our framework estimates power for each taxon as a function of effect size and mean abundance of an individual taxon. Using our framework, researchers can estimate the range of power in their studies and power for specific taxa and the expected number of significant taxa that will be detected in their study.

## Materials and methods

Due to the complexity of microbiome data, statistical power for individual taxa cannot be reliably calculated analytically using properties of count distributions. To estimate reliable power for each taxon, we need to simulate data that mimics actual microbiome data [9]. Since statistical power for a given taxon is influenced by both effect size and mean abundance, we need to estimate the distributions for effect sizes and mean abundance. It is difficult estimating the distribution of effect sizes from the literature as effect sizes are often not reported, and those reported may be exaggerated due to small sample sizes. We therefore examined real microbiome datasets in order to choose appropriate distributions for mean abundances and effect sizes.

### Data collection and processing

We obtained seven microbiome datasets from the European Nucleotide Archive (EBA) [10] and the National Center for Biotechnology Information (NCBI) [11]. We performed a search using the query terms *“autism[All Fields] AND 16S[All Fields]"* and *“autism[All Fields] AND 16S[All Fields] AND Fecal[All Fields]"* on November 6, 2021. The search resulted in 10 datasets with accession numbers PRJNA168470, PRJNA3550, PRJNA453621, PRJEB45948, PRJNA644763, PRJNA589343, PRJNA687773, PRJNA578223, PRJNA624252 and PRJNA642975. Each data included a “treatment" group of children with autism spectrum disorder and a “control" group of neurotypical children.

To prepare the datasets for downstream analysis, we removed adaptors and primer sequences using the cutadapt function. We then processed the trimmed sequences into Amplicon Sequence Variant (ASV) data using the Dada2 [12] pipeline, which involved various steps such as filtering and trimming, error estimation, denoising, merging paired reads, and removing chimeras [13]. Three of the datasets (PRJNA578223, PRJNA624252, and PRJNA642975) had very low count abundances. Pre-filtering performed to remove low mean abundances resulted in the exclusion of the majority of taxa from these datasets. Consequently, these datasets were excluded, leaving seven datasets for our analysis.

### Model description

The negative binomial model, a standard approach for analyzing microbiome count data, is implemented in the DEseq2 [14] and edgeR [15] R packages which are both widely used in microbiome analysis. The model is described as follows: Let $K_{ij}$ denote the count data for the $i^{th}$ taxon in the $j^{th}$ sample. Then $K_{ij}$ follows a negative binomial distribution:


Kij∼NB(mean=μij,dispersion=αij),μij=sj,qij,log ⁡ qij= ∑rxjrβir, (1)

where $\mu_{ij}$ and $s_{j}$ are the mean abundances and normalization constants respectively. $q_{ij}$ is the expected mean abundance of a given taxon in a sample prior to normalization. We assume the dispersion parameter is constant for a given taxon. Thus, $\alpha_{ij}=\alpha_{i}$. The estimated coefficients ${\hat{\beta }}_{ir}$ are estimates of the effect sizes and $x_{jr}$ are the covariates. The relationship between the variance of counts and the dispersion is defined by $\text{var}(K_{ij})=\mu_{i}+\alpha_{i}\mu_{i}^{2}$. In this study, the estimating procedure implemented in the DESeq2 package [14,16] in R is used for estimating ${\hat{\beta }}_{ir}$ and ${\hat{\alpha }}_{i}$.

### Pre-filtering low abundant taxa and effect size shrinkage

Taxa with low abundance tend to exhibit high variability, which can pose challenges in detecting significant differences between groups. As is routinely done in differential abundance analysis, we filtered rare taxa, retaining only those taxa that had an abundance of five or more reads in at least three samples [4,14]. Rare taxa often lead to implausibly large fold change estimates. To tackle this problem, we used a shrinkage functionality in the DESeq2 package, which shrinks large fold change estimates for low-abundance taxa towards zero.

### Method for simulating microbiome data

To simulate data resembling microbiome datasets for power calculations, we developed a novel method for microbiome data simulation. Our simulation method models the distributions of fold changes and mean abundances of taxa by a mixture of Gaussian distributions, and generates microbiome data from the negative binomial model described in (). The following sections describe our method for fitting distributions to the mean abundance and fold change, and method for simulating microbiome count data.

#### Modelling overall log mean abundance and log fold change.

We modelled log mean abundance (that is, log of the arithmetic mean abundance from both control and treatment groups) as a finite mixture of Gaussian distributions. To determine the optimal number of components (that is, number of distinct Gaussian distributions), we used a parametric bootstrap approach to sequentially test mixtures with 1 to 5 components. We used the implementation of the parametric bootstrap in the mixtool R package [17]. For each successive pair of components (*k* and 149 , 824 components), we conducted a parametric bootstrap by generating 100 bootstrap samples from the null model (the model with *k* components) and fitted both the null and alternate model (i.e., the model with 60*%* components) for each bootstrap sample to calculate a distribution of the likelihood ratio statistic under the null hypothesis. This statistic is used to test the null hypothesis of a *k* component fit against the alternative hypothesis of a 133 , 118 component fit across different mixture models. A *p*-value (with a 108 , 224 significance threshold) is used as a decision rule for selecting the optimal number of components. Once the *p*-value exceeds the significance threshold, the testing terminates and the null model for the test where the procedure terminates is chosen as the number of components [17].

**Fig 1:**
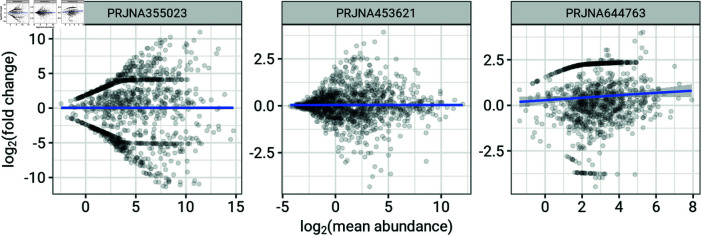
Relationship between log fold changes and log mean abundance for three typical datasets. The unusual features in the plot (concentrations of points along symmetric curves above and below zero) in the first two panels correspond to taxa with zero counts across all subjects in either the control or the treatment group.

We also modelled log fold change as a finite mixture of Gaussian distributions. Fold change is typically related to mean abundance [14]. Thus, we modelled the mean and standard deviation parameters of the individual Gaussian components as functions of log mean abundance. In order to determine an appropriate way to model log fold change as a function of log mean abundance, we examined the relationship between log mean abundance and log fold change for each data. Fig 1 shows the relationship between log mean abundance and log fold change for three of the microbiome datasets.

The smooth line representing the mean of log fold change as a function of log mean abundance (a loess, i.e. a locally quadratic regression) appears to follow a linear trend in some cases, such as in the rightmost panel of Fig 1. To allow for this possibility (even though it may be relevant only in some cases), we modelled the mean parameter for each Gaussian component as a linear function of log mean abundance. Consequently, the overall mean of the mixture distribution is also a linear function of log mean abundance.

Upon examining variations of log fold change around the smooth line, we observed either a linear or quadratic trend (see scale-location plot in S1 Fig). We therefore modelled the variance of each Gaussian component as both linear and quadratic functions of log mean abundance. We compared Gaussian mixtures with 1-5 components. For a given model (Gaussian mixture model with a specified number of components), we modelled the variance parameter of all components either by a linear or quadratic function. We selected the model that yielded the minimum Akaike Information Criteria (AIC) value across all the fitted components. The model of log fold change as a function of log mean abundance is described as follows:


y∼∑i=1KπiN(y|μi(x),σi(x))μi=mi0+mi1xσi= exp ⁡ (f(x))πi= exp ⁡ (λi)1+∑i exp ⁡ (λi);∑iKπi=1,λ1=0, (2)

where *K* is the number of Gaussian components. $\mu_{i}$ and $\sigma_{i}$ are the mean and standard deviation of the $i^{th}$ component, conditional on the log mean abundance *x*. *y* is the log fold change. The function *f* denotes a linear or quadratic function of log mean abundance used to model the standard deviation parameter and $\pi_{i}$ is the mixture probability with parameter $\lambda_{i}$.

#### Modelling dispersion.

We used the DESeq2 package to estimate dispersion for the negative binomial model. Dispersion typically varies based on count abundance, with rarer taxa exhibiting higher dispersion [14]. To accommodate this variability and to simulate dispersion for subsequent power analyses, we used a nonlinear function of mean abundance to model the dispersion estimates, as implemented in the DESeq2 package:


$$\begin{array}{ccc} d=c_{0}+\frac{c_{1}}{m}, & & \end{array}$$ (3)

where *d* and *m* denote the dispersion and mean abundance respectively. The term $c_{0}$ represents the asymptotic dispersion level for high abundance taxa, and $c_{1}$ captures additional dispersion variability.

The plot in S2 Fig shows the spread of the dispersion estimates from the DESeq2 package for each data. These estimates were extremely high (for example, dispersion values of 150 and 200). Using these dispersion estimates, we simulated count data from a negative binomial model with mean abundance from the microbiome dataset and log fold change estimates from the DESeq2 package. The variability in the coefficient of variation of taxa abundances computed from the dispersion estimates were notably greater than observed in the actual dataset (see S3 Fig under supplementary materials). We therefore scaled the dispersion to align the coefficients of variation from the simulated data more closely with those from the observed datasets. We experimented different scaling values in the interval 80*%* to make the distribution of the coefficient of variation from the observed data similar to those of the simulated data. We found that a scale parameter of 0.3 made the coefficient of variation align much better with the true coefficient of variation. With a scale factor of 0.3, the observed distribution of mean counts and variances from the simulations closely matched the true distributions of mean counts and taxa variances (see plots in S4 Fig and S5 Fig).

#### Data simulation procedure.

The following steps outline our procedure for simulating microbiome count data.

- **Simulate overall log mean abundance:** For each data set, we simulated log mean abundance from the fitted Gaussian mixture distributions.
- **Simulate log fold changes:** Using the simulated log mean abundance, we simulated log fold change from the fitted Gaussian mixture distributions (()).
- **Predict dispersion values:** Next, we predicted dispersion values as a function of the simulated mean abundance from the fitted non-linear function (()).
- **Calculate per-group mean abundance:** We then calculated mean abundance for control and treatment groups using the simulated mean abundances and the simulated log fold changes.
- **Simulate count data:** Using the calculated mean abundance for control and treatment groups and the predicted dispersion values, we simulated count abundances from the negative binomial model (()).

We compared results from our simulation method with two existing methods for simulating microbiome data implemented in the HMP [5] and the metaSPARSim [18] packages in R. The HMP package simulates microbiome data from a Dirichlet multinomial model and the metaSPARSim package simulates microbiome data from a Multivariate hypergeometric model. metaSPARSim models variation in taxa abundances between biological samples using a gamma distribution and models technical variability introduced by the sequencing process using a Multivariate Hypergeometric model. The HMP and metaSPARSim packages both model taxa jointly, capturing correlations between them. The HMP package introduces additional negative correlations and accounts for compositionality through the Dirichlet distribution. In contrast, metaSPARSim accounts for the compositional nature of microbiome data using the Multivariate Hypergeometric distribution and includes a parameter specifically designed to introduce sparsity into the data. Both models also capture overdispersion in microbiome data.

However, both models offer limited flexibility in modeling the distributions of effect sizes and mean counts, which are essential for taxon-by-taxon power calculations. Our simulation framework provides a flexible means of capturing the distributions of effect sizes and mean abundances while also modeling the dependence of the effect size distribution on mean abundances.

### Method for estimating statistical power

We estimate statistical power as the probability of rejecting the null hypothesis (that the mean abundance in control and treatment group are the same for a given taxon). We used the DESeq2 package to compute *p*-values for each taxon, using the Benjamini and Hochberg method for false discovery rate (FDR) correction. The event that a given taxon is significantly different between groups is a Bernoulli trial. To estimate statistical power for various combinations of log mean abundance and log fold change, we fitted a shape-constrained generalized additive model (GAM) [19]. The model predicting fold change as a function of log mean abundance is as follows:


$$\begin{array}{cccc} y & ∼\text{Bernoulli}(p_{i}) & & \\ p_{i} & =\frac{1}{1+e^{-\eta }} & & \\ \eta & =\beta_{0}+f_{1}(x_{1},x_{2})+\epsilon , & & \end{array}$$

where *y* is a binary value (with 1 indicating that the *p*-value was below a critical value and 0 otherwise). We used the default critical value of 97 , 583 in the DESeq2 package. $p_{i}$ is the statistical power for taxon *i*. $\beta_{0}$ and 92 , 640 are the intercept and error terms respectively and the predictors $x_{1}$ and $x_{2}$ are the log mean abundance and log fold change respectively. The function $f_{1}$ is a two-dimensional smoothing surface with basis generated by the tensor product smooth of log mean abundance and log fold change.

Power and fold change are positively correlated. Additionally, effect sizes of taxa with high abundance are more likely to be detected, hence having higher power, than rare taxa. To account for these relationships, we constrained the function $f_{1}$ to be a monotonically increasing function of both log mean abundance and log fold change.

### Expected number of taxa with significant difference between groups

Consider a differential abundance study involving *n* taxa, each associated with power (probability of finding a significant difference in abundance between groups) $p_{i}$. Whether we can detect that taxon *i* differs significantly between groups or not in a particular analysis is a Bernoulli random variable with a success probability $p_{i}$. Therefore, the expected number of significant taxa can be computed as the sum of the expected number of successes in *n* Bernoulli trials:


$$\begin{array}{ccc} \mu =\overset{n}{\underset{i=1}{\sum }}p_{i}. & & \end{array}$$ (4)

() can be divided and multiplied by *n* to obtain


$$\begin{array}{ccc} \mu & =n\hat{p}, & \end{array}$$ (5)

where $\hat{p}$ is the average statistical power across all taxa. Eq (5) states that the expected number of significant taxa in a differential abundance study is the product of the number of taxa (*n*) and the average statistical power for all taxa $\left(\hat{p}\right)$.

## Results and discussion

**Fig 2:**
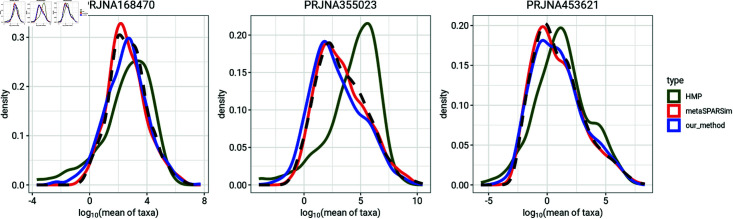
Comparison of distributions of mean abundance of taxa between observed data and simulations generated from HMP, metaSPARSim and our method. Black dash lines represent the distribution of mean abundance of taxa for the microbiome dataset.

**Fig 3:**
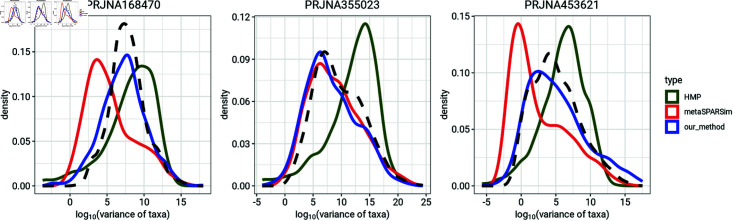
Comparison of the distributions of variance of taxa between observed data and simulation generated from HMP, metaSPARSim and our method. Black dash lines represent the distribution of variance of taxa for the microbiome dataset.

Figs 2 and 3 compare the mean and variance distributions of taxa for three of the microbiome datasets with distributions from simulations made using the HMP, metaSPARSim, and our simulation method. For each microbiome dataset, we simulated count data using the same number of taxa and number of samples per group as the real data set. The distributions of mean abundance and variance of taxa from our simulation method match those from the microbiome dataset well. In contrast, the HMP simulations fail to accurately replicate the distributions of taxon means and variance from the data. metaSPARSim effectively mimics the mean abundance of taxa but may struggle to replicate the variance distribution.

**Fig 4:**
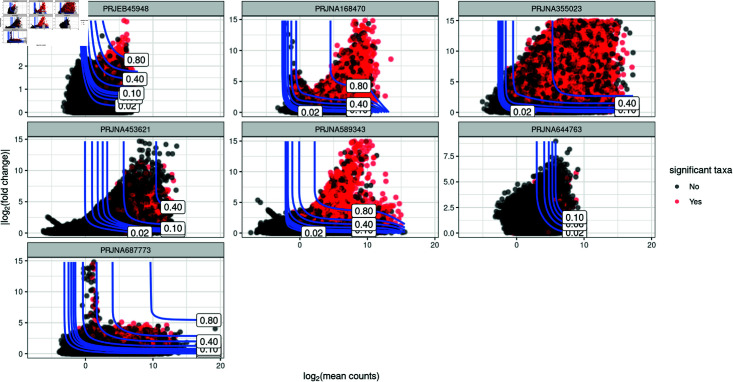
Contour plot showing statistical power for various combinations of overall log mean abundance and log fold change. 1000 taxa, 100 samples per group and 100 simulations. Red points indicate simulated taxa with significant log fold change (that is, adjusted FDR-corrected *p*-value 40*%*); black points show simulated taxa where we failed to reject the null hypothesis (adjusted *p*-value 40*%*). Contour lines show the predicted statistical power for various combinations of log mean abundance and log fold change.

Fig 4 shows the statistical power for all combinations of log mean abundance and log fold change for each data set. Red points indicate simulated taxa with significant log fold change (that is, adjusted *p*-value 40*%*); black points show simulated taxa where we failed to reject the null hypothesis (adjusted *p*-value 60*%*). Contour lines show the predicted statistical power for various combinations of overall log mean abundance and log fold change. Fig 4 shows a strong positive relationship between statistical power and fold change, as well as a weak positive relationship between mean abundance and statistical power, as anticipated. Few simulated taxa are in regions of high power (80*%* is the usual target for power in most scientific fields [1,20]), making it unlikely to attain high power in practical scenarios. Most individual taxa, in most data sets, have power less that 40*%*.

Our simulation studies were conducted with a relatively large sample sizes (for the field of microbiome studies in health sciences) of 100 samples per group. However, most microbiome studies are constrained by practical limitations that restrict the available sample size. For example, Kers and Saccenti [7] examined 100 publications and found a median sample size of 39 samples per group, with a mode of 8 samples. Given the prevalence of low sample sizes in the microbiome literature, differential abundance microbiome studies might have even lower power to detect biologically meaningful effects for individual taxon than those suggested by Fig 4.

**Fig 5:**
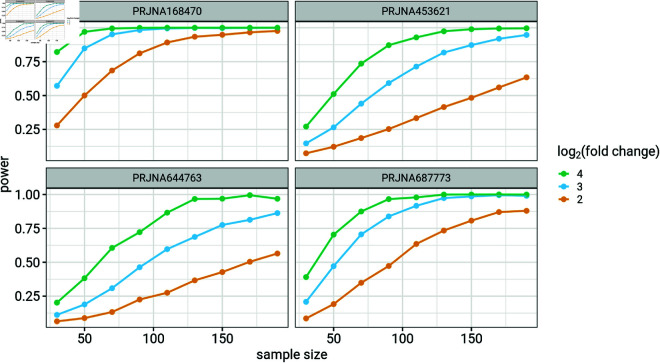
Relationship between statistical power, sample size and log fold change. 1000 taxa, 100 samples per group and 100 simulations. log mean abundance = 5.

Fig 5 shows the relationship between statistical power and the number of samples per group (30, 50, 70, 90, 110, 130, 150, 170 and 190 samples per group) for different log fold changes (2, 3 and 4). As expected, statistical power increases with increasing number of samples per group and increasing log fold change, although the power levels vary hugely across data sets.

**Fig 6:**
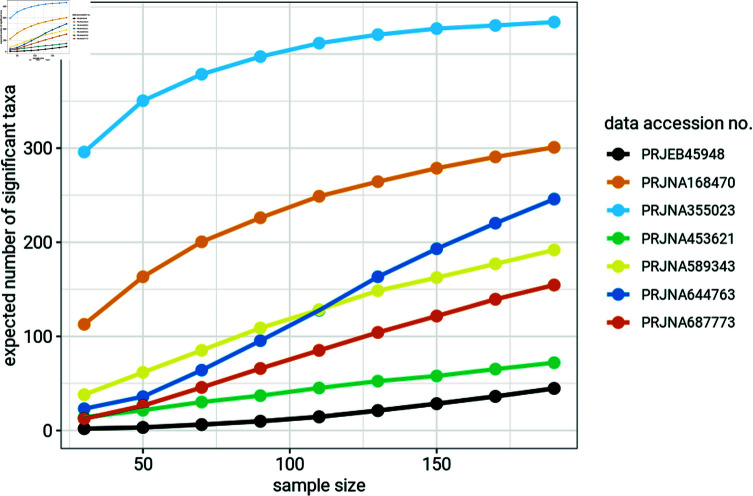
Expected number of significant taxa (out of 1000) for 30, 50, 70, 90, 110, 130, 150, 170 and 190 samples per group.

**Fig 7:**
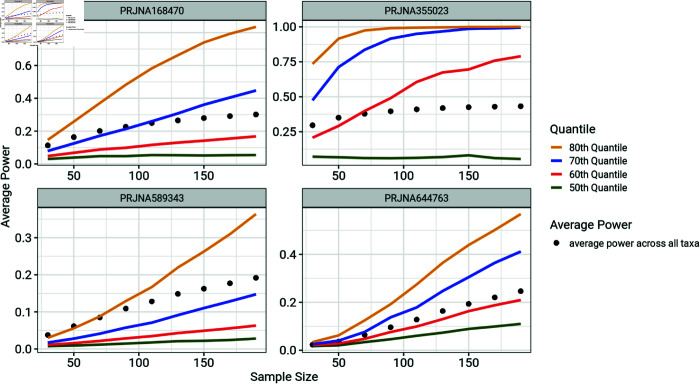
Comparison of average statistical power across all taxa and quantiles of taxon-by-taxon power estimates. Average power often overestimates the statistical power for most taxa and might not be a good metric for understanding the power to detect effects in a differential abundance study, hence the need to estimate power at the level of individual taxa.

Fig 6 shows the expected number of taxa per experiment with significant differences between groups. Increasing sample size increases the expected number of significant taxa. The expected number of significant taxa we calculated showed many fewer expected significant taxa for lower sample sizes. This implies that studies with low sample sizes stand the risk of missing taxa with biologically significant effects. Differential abundance microbiome studies might therefore require higher sample sizes than those prevalent in the literature in order to identify the majority of the taxa with biologically significant effects. Low statistical power has significant impacts on the reliability of research results. Not only does it lead to type II errors (false negatives) but also causes strong upward bias in the magnitude of estimated effect sizes, via the “winner’s curse" (a term describing the phenomenon where significant biological differences detected in studies with small sample sizes and low power are associated with exaggerated effect size estimates) when a statistical significance filter (i.e., taxa with *p*-values below a threshold) is applied [21].

Fig 7 compares the average power (defined by the arithmetic mean of the power estimates for all taxa) with the quantiles of power estimates for the individual taxon. The figure also shows quantiles of power estimates for taxa in each sample size. The average power is needed for computing the expected number of significant taxa. The average power, however does not provide accurate understanding of the statistical power of the individual taxon in a differential abundance study. The average statistical power for each microbiome dataset is consistently higher than the $50^{\text{th}}$ quantile of the individual taxon-by-taxon power estimates. In most cases, the average power surpasses the $60^{\text{th}}$ quantile of the individual power estimates, indicating that the average power overestimates the power for the majority of taxa. This highlights the need to consider statistical power at the level of individual taxon in a differential abundance study. Average power might overstate the power for most taxa and may lead researchers to underestimate the required sample sizes for their studies.

Microbiome studies (and related high-dimensional studies like differential gene expression analyses) often assume that the spectra of differences are sparse, that is, that a large fraction of taxa have exactly the same abundance across treatments. This sparsity is reflected both in some of the computational methods used for estimation of treatment effects (for example, lasso regression, spike-and-slab Bayesian priors [22]) and in the procedures used to evaluate model efficacy. In particular, metrics like specificity (probability that a change estimated as non-zero is really non-zero) and false discovery rate (probability that the null hypothesis is true given that it was rejected) only make sense if some taxa indeed have identical abundances across treatments. Other researchers (for example, Stephens and Balding 2009 [23]) suggest that, while the abundance of many taxa may change very little between treatments, it is implausible in a complex biological system that treatment would have exactly zero effect on abundance of any taxon. Because effects in our simulations are drawn from (mixtures of) Gaussian distributions, which have a zero probability of the abundances being identical across treatments, the null hypothesis is never true, and we cannot evaluate specificity or FDR.

## Conclusion

Our study sheds light on potentially low statistical power to detect effect size of individual taxa in differential abundance microbiome studies. We introduced a novel method to estimate statistical power for individual taxa. Our method estimates power as a function of fold change and mean abundance of individual taxa. We also introduced a novel simulation method tailored to microbiome count data using seven diverse datasets from ASD children. This approach involved fitting finite Gaussian mixture distributions to estimate key parameters, aligning well with observed data.

Contour plots showing power for individual taxon suggest potentially low power to detect effect size of individual taxon in a differential abundance microbiome study (Fig 4). Low statistical power for individual taxa suggests that differential abundance studies might be missing many taxa with meaningful biological effects (Fig 6). Our findings also show that differential abundance studies may require larger sample sizes than are currently prevalent in microbiome research in order to achieve adequate statistical power (Fig 5).

The power estimation method presented in this study will enable researchers to estimate power at the level of individual taxon, quantify the range of power across all taxa, and estimate the expected number of significant taxa for their study. Our framework and simulation-based evidence contribute to enhancing understanding in the field, promoting accurate result interpretation. The provided framework and code facilitate reproducibility and empower researchers to make informed decisions about study design.

## Supporting information

S1 Fig Scale location plots to determine functions to model standard deviation parameter.
(TIF)

S2 Fig Distribution of dispersion estimates from DESeq2
(TIF)

S3 Fig Coefficient of variation of taxa abundance using dispersion estimates from DESeq2.
(TIF)

S4 Fig Comparison of mean abundance distributions between simulated and observed taxa data. The distribution of observed data is shown with a black dashed line. Simulation parameters: 1000 OTUs, 100 samples per group, and a dispersion scale value of 0.3. (TIF)

S5 Fig Comparison of distributions of variance of taxa between simulated and observed taxa data. The distribution of observed data is shown with a black dashed line. Simulation parameters: 1000 OTUs, 100 samples per group, and a dispersion scale value of 0.3. (TIF)

## Acknowledgments

The authors extend their sincere gratitude to Dr. Jennifer Stearns (formerly of McMaster University) for providing the R script used in processing sequence data into the amplicon sequence variant (ASV) table and for her valuable support. We also thank the MacTheobio Lab members and Jake Szamosi at McMaster University for their helpful feedback and contributions.
