# Supplementary figures and images for: Investigating statistical power of differential abundance studies
Source: PLoS One. 2025 Apr 8;20(4):e0318820. doi: 10.1371/journal.pone.0318820 (PMC11978113; doi:10.1371/journal.pone.0318820)

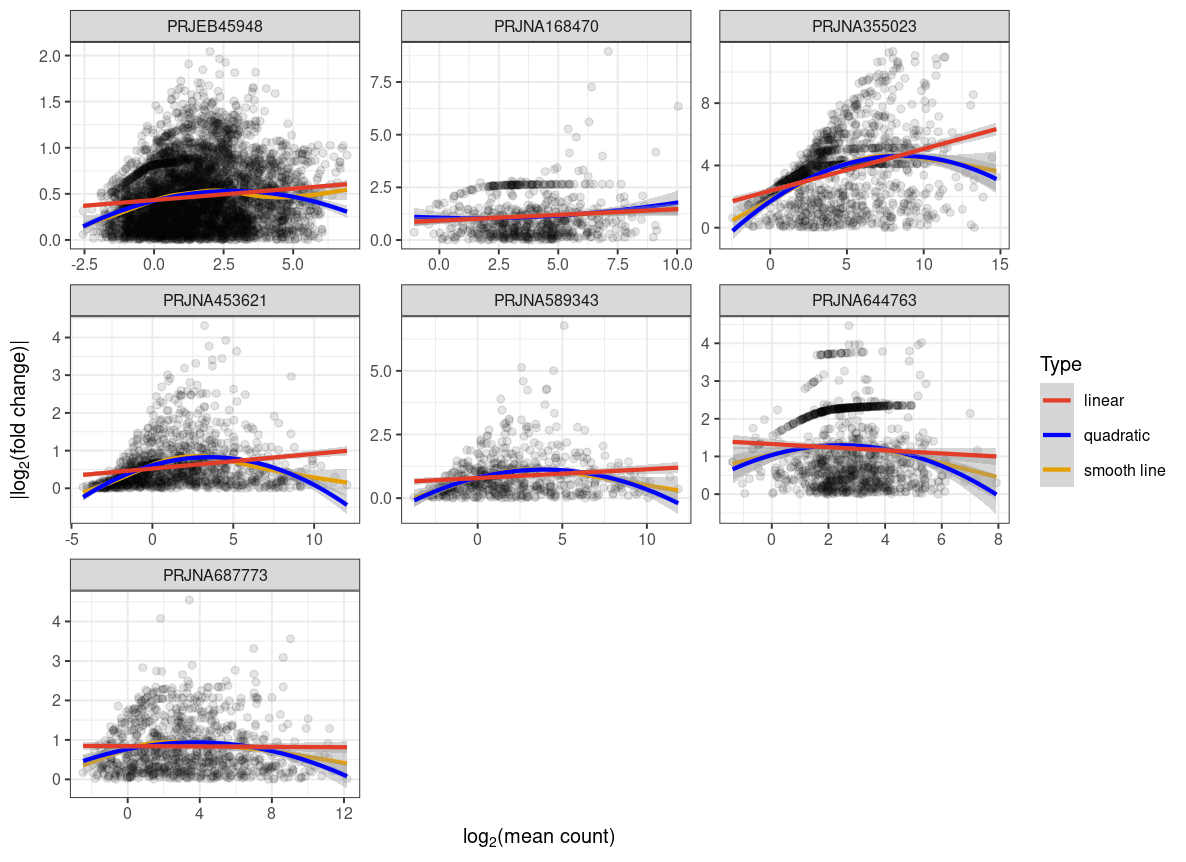

Supplement: S1 Fig — (TIF) [file pone.0318820.s001.tif]

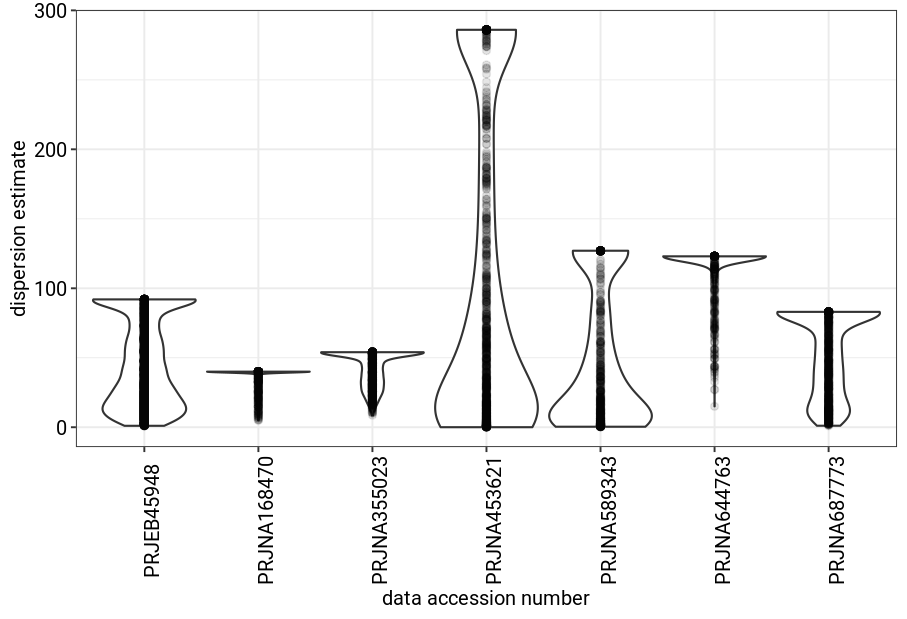

Supplement: S2 Fig — (TIF) [file pone.0318820.s002.tif]

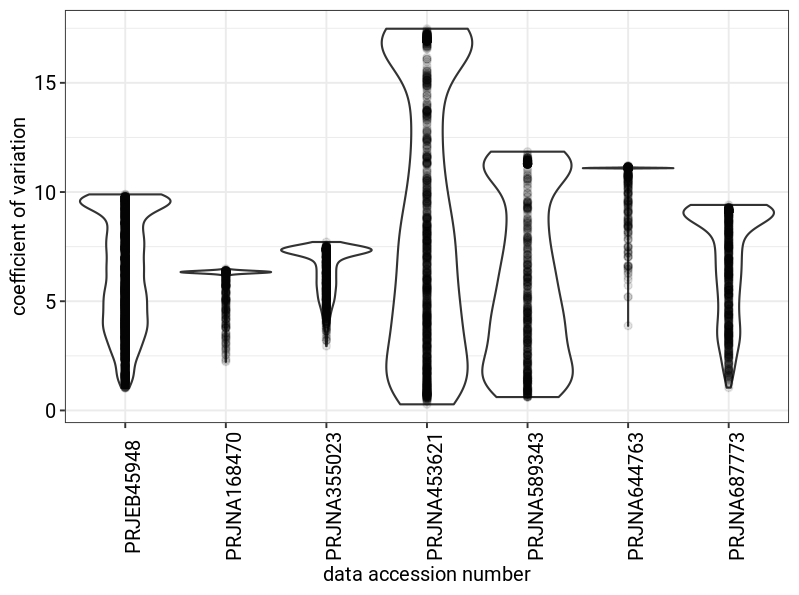

Supplement: S3 Fig — (TIF) [file pone.0318820.s003.tif]

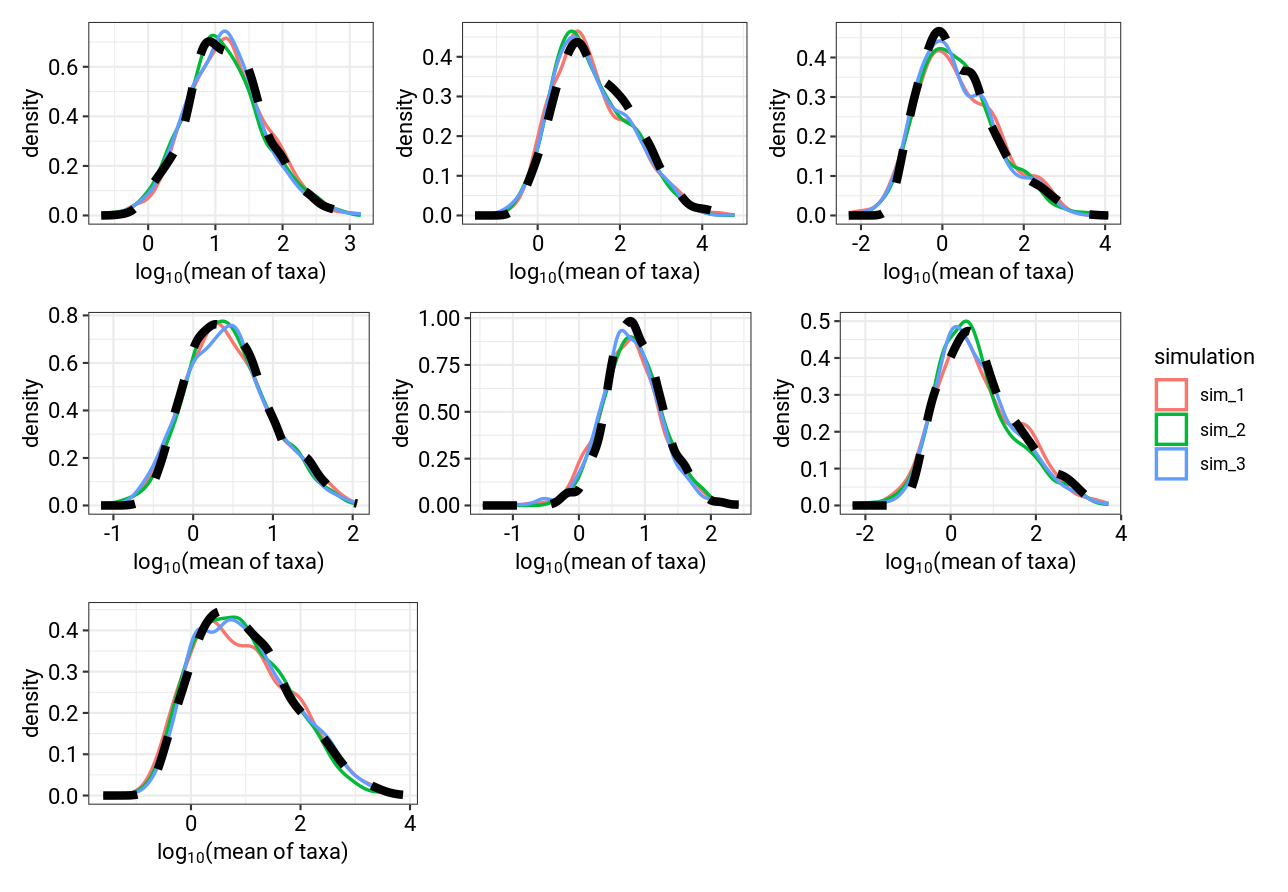

Supplement: S4 Fig — (TIF) [file pone.0318820.s004.tif]

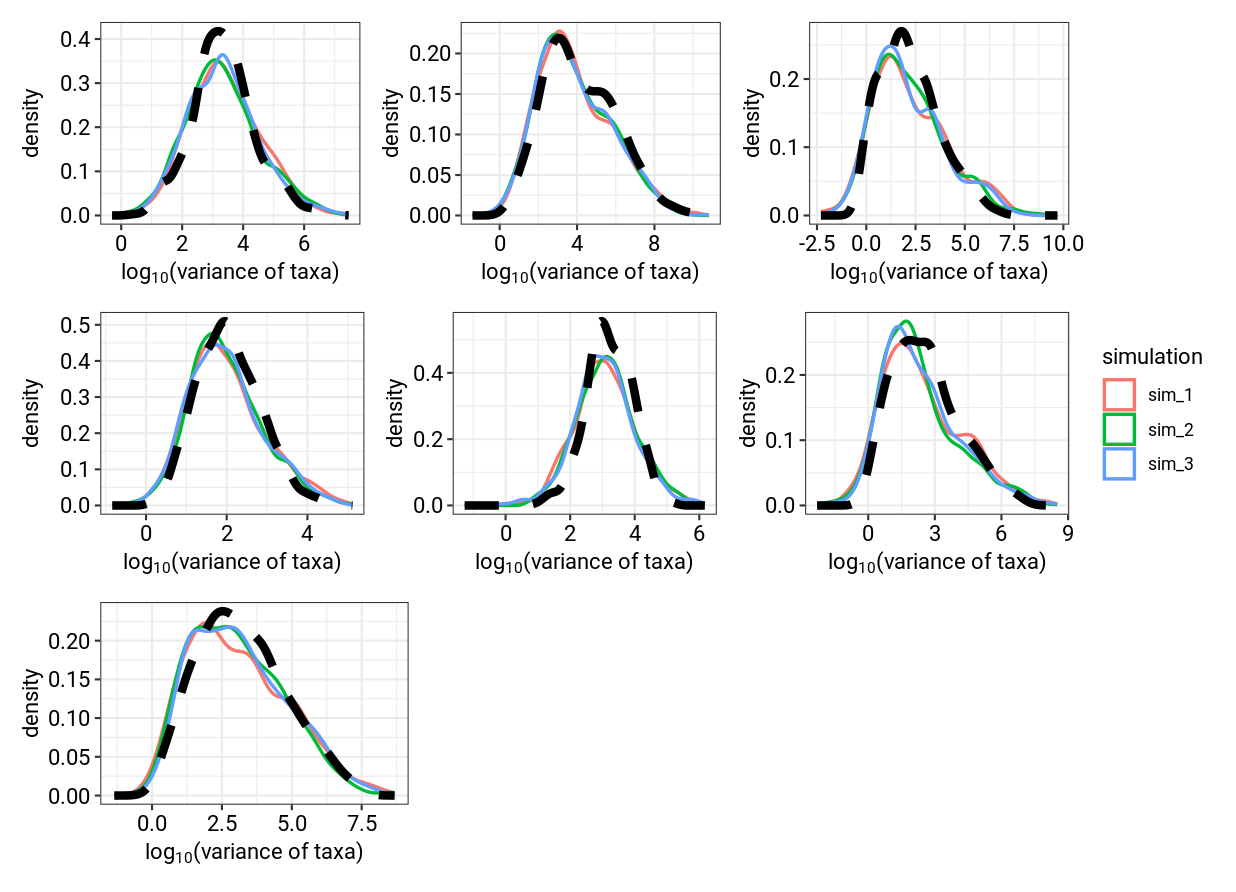

Supplement: S5 Fig — (TIF) [file pone.0318820.s005.tif]
